# Endoplasmic reticulum stress-induced apoptosis in intestinal epithelial cells: a feed-back regulation by mechanistic target of rapamycin complex 1 (mTORC1)

**DOI:** 10.1186/s40104-018-0253-1

**Published:** 2018-05-03

**Authors:** Yun Ji, Xuan Luo, Ying Yang, Zhaolai Dai, Guoyao Wu, Zhenlong Wu

**Affiliations:** 1 State Key Laboratory of Animal Nutrition,Department of Animal Nutrition and Feed Science, China Agricultural University, Beijing, 100093 China; 2 Department of Animal Science, Texas A&M University, College Station, TX USA

**Keywords:** Apoptosis, Barrier function, ER stress, Intestinal epithelial cell, mTORC1

## Abstract

**Background:**

Endoplasmic reticulum (ER) stress is associated with multiple pathological processes of intestinal diseases. Despite a critical role of mechanistic target of rapamycin complex 1 (mTORC1) in regulating cellular stress response, the crosstalk between mTORC1 and ER stress signaling and its contribution to the intestinal barrier function is unknown.

**Results:**

In the present study, we showed that intestinal epithelial cells (IEC-6) incubated with tunicamycin led to caspase-3-dependent apoptotic cell death. The induction of cell death was accompanied by activation of unfolded protein response as evidenced by increased protein levels for BiP, p-IRE1α, p-eIF2α, p-JNK, and CHOP. Further study demonstrated that tunicamycin-induced cell death was enhanced by rapamycin, a specific inhibitor of mTORC1. Consistently, tunicamycin decreased transepithelial electrical resistance (TEER) and increased permeability of the cells. These effects of tunicamycin were exacerbated by mTORC1 inhibitor.

**Conclusions:**

Taken together, the data presented here identified a previously unknown crosstalk between an unfold protein response and mTORC1 signaling in the intestinal epithelium. This feed-back loop regulation on ER stress signaling by mTORC1 is critical for cell survival and intestinal permeability in epithelial cells.

## Background

The endoplasmic reticulum (ER) is the main organelle associated with the processing and folding of newly synthesized proteins before they are transported to other cellular components [1]. Various cellular stress conditions can result in the accumulation of unfolded or misfolded proteins in the ER lumen, which might trigger an unfolded protein response (UPR). UPR is an integrated mechanism that allows the cells to cope with the unfolded/misfolded proteins and therefore maintaining cellular homeostasis by increasing the folding capacity of the ER, inhibiting protein synthesis, and promoting the degradation of unfolded proteins [2]. In mammalian cells, three protein sensors responsible for the initiation of the UPR have been identified on the ER membrane: inositol-requiring enzyme 1 (IRE1), protein kinase RNA-like ER kinase (PERK), and activating transcription factor 6 (ATF6). In the absence of ER stress, the luminal domains of the ER protein sensors are bound in an inactive state by BiP, an ER chaperone binding immunoglobulin protein also known as glucose-regulated protein 78 (GRP78) [3]. Accumulation of unfolded/misfolded proteins dissociates BiP from the luminal domains of the protein sensors, resulting in activation of UPR signaling [4]. The UPR is an adaptation response that is conducive to cell survival by reducing the accumulation of unfolded proteins and restoring an appropriate ER function. However, prolonged or severe ER stress activates and initiates apoptosis signaling [5].

As the predominant cells that line the gastrointestinal tracts, intestinal epithelial cells are constantly exposed to various environmental factors, such as intestinal microbiota, dietary components, nutrient metabolites, and toxins, which can trigger ER stress and consequently lead to intestinal mucosal barrier dysfunction [6, 7]. Accumulating evidence indicates that ER stress in epithelial cells is associated with pathogenesis of intestinal disorders, such as colitis and inflammatory bowel disease [7–10]. Importantly, the UPR signaling is essential for maintenance of the intestinal homeostasis [10]. Genetic deletion of genes involved in the UPR is associated with increased intestinal inflammation and integrity breakdown, as well as apoptosis [11, 12]. Despite much progress in the understanding of the implications of ER stress in intestinal disorders, the regulation of ER stress in intestinal epithelial cells remains largely unknown [13]. It has been reported that the intestinal barrier integrity can be disrupted by ER stress-related apoptosis; however, the underlying mechanisms remain elusive.

The mechanistic target of rapamycin (mTOR) is a master regulator of various cellular processes including cell proliferation, survival, metabolism, autophagy, and the synthesis of proteins and lipids [14]. The two complexes of mTOR, complex 1 (mTORC1) and complex 2 (mTORC2), execute distinct cellular responses due to their different subunit compositions [15]. The mTORC1 signaling is mainly activated by a variety of intracellular and extracellular stimuli, for example, nutrients, growth factors, and stress [15, 16]. Recent studies showed that mTORC1 signaling is intertwined with the ER stress response and contributes to the cell fate decision [17–21]. Current understanding of the implications of the interplay between these two critical pathways in intestinal mucosal integrity is unknown. In the present study, IEC-6 cells were cultured in the presence or absence of tunicamycin, an ER stress inducer that inhibits N-linked glycosylation. We showed that tunicamycin exposure led to activation of UPR signaling and apoptotic cell death, as well as activation of mTORC1 signaling. Intriguingly, inhibition of mTORC1 with rapamycin resulted in increased cell apoptosis and increased intestinal permeability, suggesting a feed-back loop regulation of mTORC1 signaling on ER stress-induced apoptosis.

## Methods

### Reagents

Tunicamycin and FITC-dextran (20 kDa) were bought from Sigma Chemicals Co. (St. Louis, MO, USA). DMEM and fetal bovine serum (FBS) were bought from GIBCO BRL (Grand Island, NY, USA). Cell counting kit-8 (CCK-8) reagent was from ZOMANBIO Biotechnology Co. (Beijing, China). Hoechst 33342 was bought from Biotium (Fremont, CA, USA). TRIzon Reagent was obtained from CWBIO biotech Co. (Beijing, China). PrimeScript^®^ RT Master Mix and SYBR^®^ Premix EX Taq™ II kits were purchased from Takara Biotechnology Co. (Dalian, China). Antibodies against CHOP, Bcl-2, and β-actin were from Santa Cruz Biotechnology (Santa Cruz, CA, USA). Rapamycin and antibodies against cleaved-caspase-3, IRE1α, BiP, ATF4, c-myc, phospho-eIF2α (Ser51), eIF2α, phospho-p38 MAPK (Thr180/Tyr182), p38 MAPK, phospho-ERK1/2 (Thr202/Tyr204), ERK1/2, phospho-JNK (Thr183/Tyr185), JNK, phospho-mTORC1 (Ser2448), mTORC1, phospho-p70S6K (Thr389/Thr412), p70S6K, phospho-4EBP1 (Thr70), and 4EBP1 were obtained from Cell Signaling Technology (Beverly, MA, USA). Antibody against phospho-IRE1α (Ser274) was purchased from Abcam Inc. (Cambridge, MA, USA). Peroxidase-conjugated goat anti-rabbit and goat anti-mouse secondary antibodies were purchased from Huaxingbio Biotechnology Co. (Beijing, China).

### Cell culture and treatment

IEC-6 cells cultured with Dulbecco’s modified eagle medium (DMEM) supplemented with 10% fetal bovine serum (FBS) and 1% penicillin–streptomycin were incubated at 37 °C in a humidified incubator with 5% CO_2_. For ER stress stimulation or rapamycin treatment, the medium was replaced by DMEM containing indicated doses of tunicamycin or rapamycin for appropriate time points.

### Cell viability

Cells (10,000 cells/well) were seeded into 96-well plates and treated as indicated. 10 μL of WST-8 solution was added to each well and then was incubated for another 30 min in a 37 °C incubator. Cell viability was assessed by the production of formazan from the reduction of WST-8 by intracellular mitochondrial dehydrogenase. The absorbance at 450 nm was measured using a microplate reader (SpectraMax® M3, Molecular Devices, USA).

### Hoechst 33342 staining

Cells were seeded in 6-well plates (2 × 10^5^ cells per well) and treated as indicated followed by staining with 5 μg/mL of Hoechst 33342 for 15 min at 37 °C. Then the apoptotic morphologic features, including the condensation of nuclear DNA and reduced cell volume, were observed under a fluorescence microscope (Zeiss, Germany).

### Flow cytometry analysis

Cells were harvested with 0.25% trypsin and resuspended in 1 × binding buffer, followed with Annexin V-FITC and PI staining at room temperature for 15 min. Flow cytometric analysis was performed using a CytoFLEX flow cytometer (Beckman Coulter, USA), and data were collected by the CytExpert software (version 1.2). The percentages of apoptotic cells at early phase plus late phase were used for statistical analysis.

### Real-time PCR assay

To analyze the transcriptional abundance of genes, total RNA was extracted from cells by using the Trizol reagent and reverse-transcribed into cDNA with a PrimeScript^®^ RT Master Mix kit (TaKaRa, Japan) by following the manufacturer’s protocol. Quantitative PCR was performed with SYBR Green (TaKaRa, Japan) using the ABI 7500 real time-PCR system (Life Technologies, USA). Primer sequences for each gene are listed in Table 1. *β-actin* was used as a reference gene in the calculation of the relative expression level of a target gene by the 2^−ΔΔCT^ method [22].

**Table 1:** Primer sequences used for real-time PCR

Gene	Accession No.	Primers (5′→3′)	Product size, bp
*ATF4*	NM_024403.2	F: GCCAAGCACTTCAAACCTCA R: CAATCTGTCCCGGAAAAGGC	125
*HSPA5*	NM_013083.2	F: TGATATCGGAGGTGGGCAAA R: CTTTCCCAAATACGCCTCGG	100
*XBP1s*	NM_001004210.2	F: ACAGACTGCGCGAGATAGAA R: ATCCCCAAGCGTGTCCTTAA	165
*DDIT3*	XM_006241444.2	F: GTCACAAGCACCTCCCAAAG R: ACTGACCACTCTGTTTCCGT	115
*β-actin*	NM_031144.3	F: TGTGTTGTCCCTGTATGCCT R: CCCTCATAGATGGGCACAGT	90

### Western blot analysis

Treated cells were harvested and then were resuspended in RIPA lysis buffer (10 mmol/L Tris-HCl, pH 7.4; 150 nmol/L NaCl; 10 mmol/L EDTA; 1% NP-40; 0.1% SDS) containing protease and phosphatase inhibitors. To obtain the whole cell lysates, the lysis solution was centrifuged at 12,000 r/min for 15 min. The bicinchoninic acid (BCA) assay was performed to evaluate the protein concentrations for each sample. Total cell lysates with 25 μg of denatured protein were separated by 12% SDS-polyacrylamide gel electrophoresis and transferred to a PVDF membrane, which was subsequently blocked by 5% nonfat dry milk in Tris-buffered saline with 0.1% Tween 20 (TBS-T) buffer for 1 h at room temperature. Then the membrane was incubated with primary antibodies (1:1,000–2,000 dilution) overnight at 4 °C. After washing with TBS-T three times, the membrane was further incubated with secondary antibodies (1:5,000 dilution) for 1 h at room temperature. The detection of immunoreactive protein bands was carried out by enhanced chemiluminescence using an ImageQuant LAS 4000 mini system (GE Healthcare). The intensity of the bands was determined by Image J software (National Institute of Health, USA).

### Monolayer transepithelial electrical resistance determination

Cells were seeded into the apical side of Transwell inserts (Corning Costar, NY, USA) in 24-well plates (8 × 10^4^ cells/well). The basolateral side of an insert contained the culture medium. After 2 d, monolayer cells were incubated with treatment medium and then the values for transepithelial electrical resistance were measured by a Millicell® ERS-2 Volt-Ohm Meter (Millipore, USA). The unit area resistance was calculated by multiplying the Ohm value by the effective surface area of the filter membrane (0.33 cm^2^).

### Monolayer paracellular permeability measurement

FITC-dextran (20 kDa) (1 mg/mL) dissolved in DMEM with tested drugs was added onto the upper layer of the monolayer in the Transwell inserts. The lower layer of the insert was supplemented with treatment medium without FITC-dextran. After incubation, the medium in the basolateral side was transferred into a 96-well plate, followed by the determination of the fluorescence intensity using a microplate reader (SpectraMax® M3) at the excitation and emission wavelengths of 490 and 520 nm, respectively.

### Statistical analysis

Results were presented as means ± SEMs and analyzed by one-way or two-way ANOVA followed with Dunnett’s *t*-test. All the analyses were conducted by using the SAS 9.1 software. A value of *P <* 0.05 was considered as statistically significant difference.

## Results

### Induction of ER stress results in apoptosis in IEC-6 cells

**Fig. 1:**
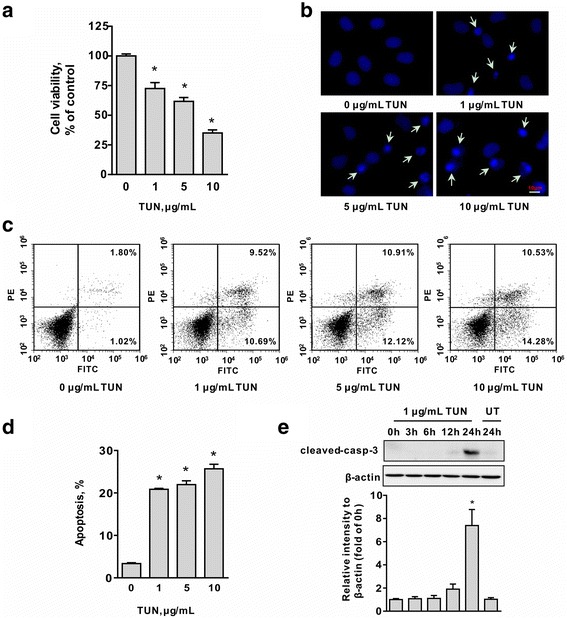
Tunicamycin treatment results in apoptotic cell death in IEC-6 cells. **a** Cells treated with tunicamycin (0, 1, 5, and 10 μg/mL) for 24 h were harvested for viability assay using a cell counting kit. Data are expressed as mean ± SEM in percentage compared with the control (0 μg/mL of tunicamycin), *n* = 6. *P < 0.05. **b** Morphological changes induced by indicated concentrations of tunicamycin (0, 1, 5, and 10 μg/mL) in IEC-6 cells stained with Hoechst 33342. The images were acquired by using a fluorescence microscope. Scale bar: 10 μm. **c** Flow cytometry analysis of apoptosis in IEC-6 cells by Annexin V-FITC/PI double staining. Cells in the right lower and right upper quadrant were considered as the early and late apoptotic cells, respectively. **d** The bar chart represents the percentage of apoptotic cells. Data are shown as mean ± SEM, *n* = 3. *P < 0.05. **e** Western blot results for the protein levels of cleaved caspase-3 in IEC-6 cells treated with (1 μg/mL) or without tunicamycin for indicated time periods. β-actin was used as a loading control. The histogram represent the statistical analysis of cleaved-caspase-3 of three individual experiments. Data are expressed as mean ± SEM. *P < 0.05. TUN, tunicamycin; UT, untreatment; cleaved-casp-3, cleaved-caspase-3

As shown in Fig. 1, IEC-6 cells treated with tunicamycin (1, 5, and 10 μg/mL) for 24 h led to decreased cell viability in a dose-dependent manner (Fig. 1a) (*P <* 0.05). The reduction of viability upon tunicamycin exposure was due to increased apoptosis as shown by condensed and fragmented nuclei (Fig. 1b), and Annexin V-FITC/PI double staining positive cells (*P <* 0.05) (Fig. 1c and d). Consistent with increased cell death, tunicamycin treatment resulted in accumulation of cleaved-caspase-3, a characteristic of apoptosis, (Fig. 1e) in IEC-6 cells, indicating the implication of caspase-3-dependent apoptosis in response to ER stress. Considering that increased apoptosis was observed in IEC-6 cells treated with 1 μg/mL of tunicamycin at 24 h post-treatment, this concentration of tunicamycin was used in the following experiments.

### Tunicamycin activates ER stress signaling in intestinal epithelial cells

**Fig. 2:**
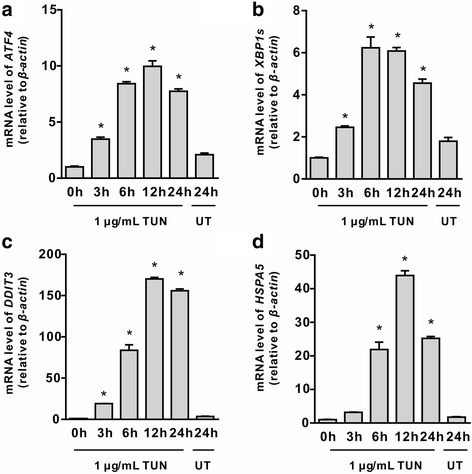
mRNA levels for ER stress-related genes in tunicamycin treated cells. IEC-6 cells were incubated with (1 μg/mL) or without tunicamycin for indicated time periods. mRNA levels for *ATF4* (**a**), *XBP1s* (**b**), *DDIT3* (**c**), and *HSPA5* (**d**) were determined by quantitative real-time PCR. *β-actin* was used as an internal reference. Data are presented as mean ± SEM, *n* = 3. *P < 0.05 by one-way ANOVA. TUN, tunicamycin; UT, untreatment

**Fig. 3:**
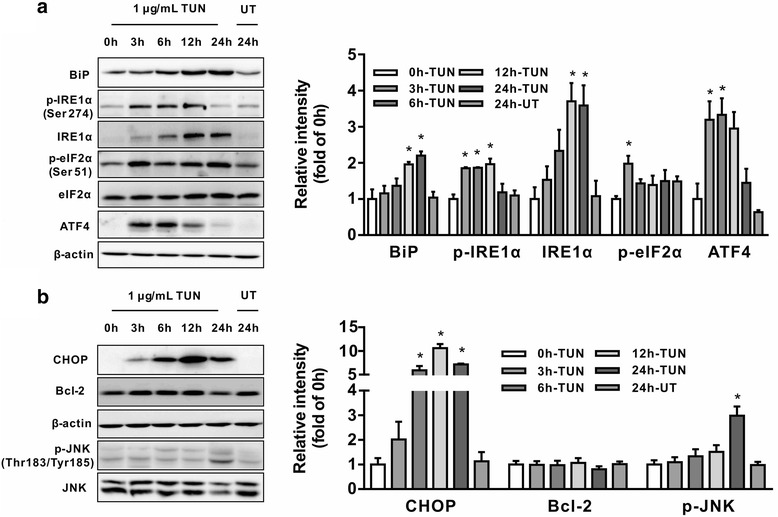
Effect of tunicamycin on ER stress pathway activation in IEC-6 cells. Cells were incubated with 1 μg/mL tunicamycin for indicated time periods, Western bolt was performed to analyze protein abundance for: (**a**) ER stress markers, including BiP, IRE-1α, p-IRE1α, eIF2α, p-eIF2α, and ATF4; and (**b**) apoptosis related proteins, including CHOP, JNK, p-JNK, and Bcl-2. The right panel histograms represent the statistical analysis of protein abundance from three individual experiments. Data are expressed as mean ± SEM. *P < 0.05. TUN, tunicamycin; UT, untreatment

To explore the involvement of UPR signaling in tunicamycin-induced cell death, we examined the expression profile of genes involved in ER stress by quantitative real-time PCR as well as Western blot analysis. Cells incubated with tunicamycin led to increased mRNA abundances for ER stress mediators (*P <* 0.05), such as *ATF4* (Fig. 2a), spliced X-box binding protein 1 (*XBP1s*) (Fig. 2b), and DNA damage-inducible transcript 3 (*DDIT3*, also known as CHOP) (Fig. 2c) at 3 h post-treatment, which were maintained until 24 h. The elevation of mRNA levels (*P <* 0.05) for heat shock 70 kDa protein 5 (*HSPA5*, the gene encoding BiP) (Fig. 2d) were observed at 6 h, and were maintained until 24 h post-treatment. Western blot analysis showed that incubation with tunicamycin activated ER stress signaling as shown by increased abundances for BiP, phospho-IRE1α, ATF4, and phospho-eIF2α (Fig. 3a) at 3 h post-treatment (*P <* 0.05). The protein abundances for phospho-IRE1α, and ATF4 were decreased at later time points (≥ 12 h) (Fig. 3a). We next determined CHOP, phospho (p)-JNK, p-p38 MAPK (p-p38), and extracellular signal-regulated kinase 1/2 (ERK1/2) proteins related to ER stress-induced apoptosis, and Bcl-2 (B-cell leukaemia/lymphoma 2), an anti-apoptotic protein. The results indicated that tunicamycin triggered activation of JNK (Fig. 3b) (*P <* 0.05), instead of p38 MAPK and ERK1/2 (Data not shown) in IEC-6 cells. Strikingly, the protein abundance for CHOP was enhanced by tunicamycin (*P <* 0.05). Tunicamycin exposure had no effect on the protein level of Bcl-2 during the time course treatment (Fig. 3b).

### Tunicamycin exposure led to activation of mTORC1 signalling

**Fig. 4:**
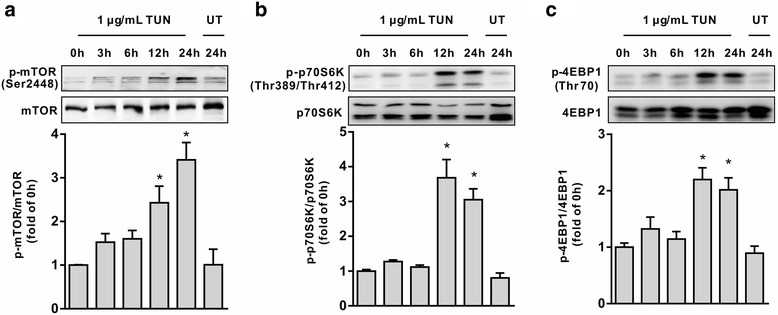
Tunicamycin induced activation of mTOR signaling. Cells were treated with or without tunicamycin for indicated time periods, protein abundance for mTORC1, p-mTORC1, p70S6K, p-p70S6K, p-4EBP1, and 4EBP1 were determined by Western blot analysis. The histograms represent the statistical analysis of protein abundance from three individual experiments. (**a**) p-mTOR/mTOR. (**b**) p-p70S6K/p70S6K. (**c**) p-4EBP1/4EBP1. Data are presented as mean ± SEM. *P < 0.05

As shown in Fig. 4, cells treated with tunicamycin led to enhanced protein abundances for phospho-mTOR, as well as downstream targets, p-4EBP1 and p-p70S6K (Fig. 4) at 12 h and 24 h post-treatment in a time-dependent manner, as compared with controls (*P <* 0.05). This result indicated an activation of mTORC1 signalling in response to ER stress in intestinal epithelial cells.

### Inhibition of mTORC1 signaling with rapamycin sensitizes cells to ER stress-associated apoptosis

**Fig. 5:**
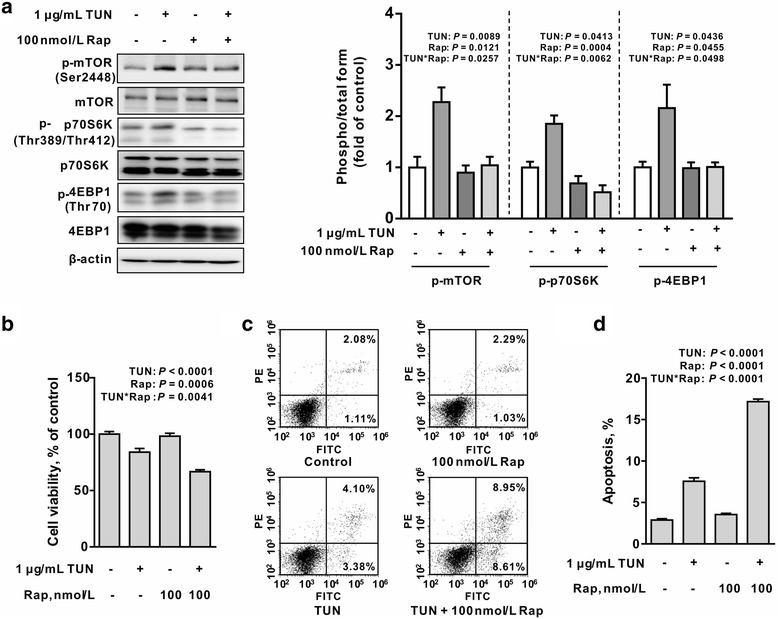
Rapamycin enhances tunicamycin-induced cell apoptosis in IEC-6 cells. **a** Rapamycin blocked tunicamycin-induced mTORC1 signaling activation. IEC-6 cells were treated with or without tunicamycin (1 μg/mL) in the presence or absence of rapamycin (100 nmol/L) for 12 h. Protein abundances for mTORC1, p-mTORC1, p70S6K, p-p70S6K, p-4EBP1, and 4EBP1 were determined by Western blot analysis. The right panel histograms represents the statistical analysis of protein abundance from three individual experiments. Data are expressed as mean ± SEM. **b** mTORC1 inhibition by rapamycin sensitized tunicamycin-induced apoptosis. Cells were treated without or with tunicamycin (1 μg/mL) in the presence or absence of rapamycin (10 or 100 nmol/L) for 12 h, cell viability was assessed by using of CCK-8 kit. Data are presented as mean ± SEM in percentage compared to the untreatment group, *n* = 6. **c** Flow cytometry analysis of apoptosis in IEC-6 cells. Cells treated as in Fig. 4c were stained by Annexin V-FITC/PI and apoptosis was determined by Facs analysis. **d** Bar chart for apoptosis in tunicamycin and/or rapamycin treated cells. Data are mean ± SEM, *n* = 3. TUN, tunicamycin; UT, untreatment; Rap, rapamycin

**Fig. 6:**
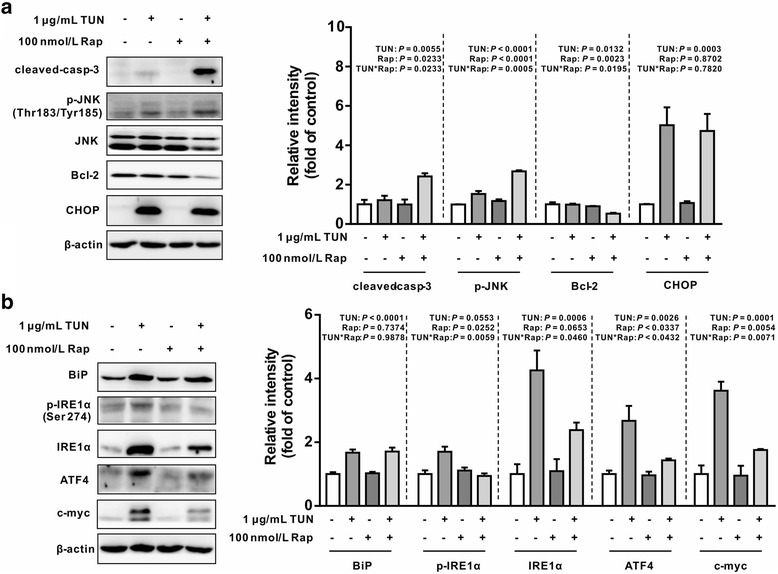
Effect of rapamycin on tunicamycin-induced ER stress markers (**a**) and (**b**) apoptosis protein abundance in IEC-6 cells. Cells were treated with or without tunicamycin (1 μg/mL) in the presence or absence of rapamycin for 12 h, protein abundance was determined by Western blot analysis. The right panel histograms at the right side of the bands represent the statistical analysis of protein abundance from three individual experiments. β-actin was used as a loading control. Data are expressed as mean ± SEM. TUN, tunicamycin; Rap, rapamycin; cleaved-casp-3, cleaved-caspase-3

mTORC1 has been reported to be implicated in and contributes to ER stress-triggered apoptosis by regulating apoptotic proteins in various cells [17, 18]. To ascertain a functional role of mTORC1 signaling in ER-stressed intestinal epithelial cells, IEC-6 cells were incubated with tunicamycin in the presence or absence of rapamycin (100 nmol/L), a specific inhibitor of mTORC1. Western blot analysis showed that tunicamycin-induced activation of mTORC1, 4EBP1 and p70S6K was significantly revered by rapamycin (Fig. 5a) (*P <* 0.05). Interestingly, we found that tunicamycin-induced apoptosis was enhanced by rapamycin as shown by cell viability and flow cytometry analysis (Fig. 5b, c and d), as compared with tunicamycin single treatment, indicating a feed-back loop regulation on ER-stress-induced apoptosis by mTORC1 activation. In agreement with phenotyptes observed, tunicamycin-induced up-regulation of JNK phosphorylation and cleavage of caspase-3, instead of CHOP, was enhanced by rapamycin as compared with tunicamycin single treatment (Fig. 6a) (*P <* 0.05). Moreover, treatment with rapamycin and tunicamycin led to reduced protein levels for Bcl-2 in cells, even though rapamycin or tunicamycin single treatment had no effect on its protein abundance. Tunicamycin-induced upregulation of phospho-IRE1α, IRE1α, and ATF4 were markedly abrogated by rapamycin (Fig. 6b) (*P <* 0.05). In contrast, rapamycin did not affect tunicamycin-induced upregulation of BiP protein levels (Fig. 6b). Interestingly, tunicamycin treatment resulted in enhanced protein levels of c-myc (*P <* 0.05), a transcriptional factor with an ability to bind to and regulate ATF4-CHOP signaling [21], which was reversed by rapamycin.

### Effects of rapamycin on intestinal permeability in tunicamycin-challenged cells

**Fig. 7:**
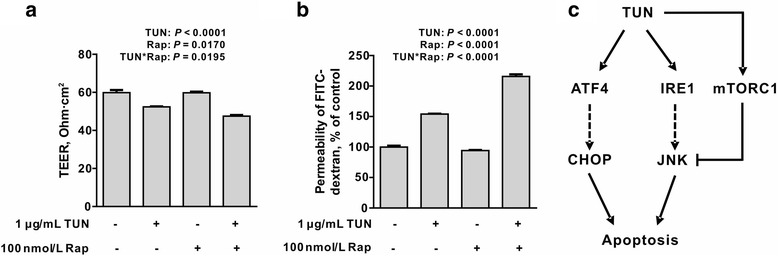
Effects of mTORC1 inhibition on intestinal barrier function in tunicamycin-challenged cells. Effect of rapamycin on tunicamycin-induced (**a**) transepithelial electric resistance (TEER), and (**b**) permeability in IEC-6 cells. IEC-6 monolayer cells incubated with tunicamycin (1 μg/mL) in the presence or absence of rapamycin (100 nmol/L) for 24 h. TEER and permeability were determined. Data are expressed as mean ± SEM, *n* = 3. TEER, transepithelial electrical resistance; FITC, fluorescein isothiocyanate; Rap, rapamycin. (**c**) Proposed mechanism for tunicamycin-induced apoptosis in intestinal epithelial cells and its feed-back regulation by mTORC1. TUN, tunicamycin

To investigate the effect of mTORC1 inhibitor on the intestinal permeability in tunicamycin-treated epithelial cells, IEC-6 cells were incubated with tunicamycin in the presence or absence of rapamycin. Compared with controls, cells treated with tunicamycin had reduced TEER (transepithelial electrical resistance) (*P <* 0.05) (Fig. 7a) and increased permeability to FITC-Dextran (*P <* 0.05) (Fig. 7b), which were further exacerbated by the mTORC1 inhibitor (*P <* 0.05). These data indicated a functional feed-back loop regulation on tunicamycin-induced intestinal permeability by mTORC1 signaling.

## Discussion

In the present study, we found that tunicamycin treatment led to caspase-3-dependent apoptosis in intestinal epithelial cells, which was accompanied by mTORC1 activation. Further study showed tunicamycin-induced apoptosis was enhanced by rapamycin, a specific inhibitor of mTORC1, indicating a feed-back loop regulation of ER stress-induced apoptosis by mTORC1 (Fig. 7c). Importantly, tunicamycin-induced impairment of intestinal permeability was exacerbated by rapamycin. These data presented here uncovered a novel interaction between mTORC1 signaling and unfolded protein response, which is critical for the intestinal integrity of epithelial cells.

ER stress is an adaptive response to stressful conditions by triggering UPR signaling under unfavorable conditions, such as hypoxia, chemo drug exposure, or nutritional deprivation. However, severe or prolonged ER stress lead to apoptotic cell death by inducing the expression of downstream genes implicated in cell death signaling pathways [23]. ER stress has been reported to be involved in intestinal disorders such as necrotizing enterocolitis [24], ulcerative colitis [25], and Crohn’s disease [26]. However, it remains largely unknown how the UPR signaling affects intestinal epithelial cells and contributes to the intestinal barrier function in humans and animals [6, 7].

In our study, IEC-6 cells derived from the rat intestinal crypt epithelium [27] were treated with different concentrations of tunicamycin, a well-known ER stress inducer [28]. Tunicamycin incubation resulted in the cleavage of caspase-3, a characteristic of apoptotic cell death which was associated with UPR signaling. This is shown by activation of ATF4 and XBP1s at both transcriptional and protein levels, as well as enhanced CHOP [24, 29] and p-JNK [30, 31], two well-known targets implicated in ER stress-related cell death. The activation of JNK and CHOP acted together, thus contributing to apoptotic cell death in intestinal epithelial cells as previously described [32]. Considering that CHOP activation has been reported to be associated with enhanced ATF6 and PERK-eIF2α-ATF4 signaling [24], more studies are required to elucidate how these two signaling pathways interact and confer to CHOP-mediated cell death effect in the intestinal epithelial cells.

A novel finding of this study is that ER stress-induced cell death occurs along with activation of mTORC1, which act as feed-back loop regulation on ER stress-induced cell death. ER stress and mTORC1 are critical signaling pathways that are associated with cell fate decision by distinct mechanisms in response to various stress insults [4, 33]. Impaired function of either ER stress or mTORC1 has been shown to be associated with deregulation of intestinal barrier function in both humans and experimental animals [1, 34]. A cross talk between these two signaling pathways was described by several lines of studies [17–20]. However, underlying mechanisms responsible for the interaction are not well defined. In line with previous study [17, 20], both mTORC1 and UPR signaling were activated by tunicamycin in intestinal epithelial cells. In contrast, we found that inhibition of mTORC1 led to markedly upregulation of p-JNK and cleavage of caspase3, downregulation of Bcl-2, increased apoptosis and decreased intestinal permeability. These results are not consistent with previous study showing that rapamycin attenuated ER-stress induced apoptosis by blocking IRE1-JNK [17], PERK-CHOP [20], or mTORC1/c-myc signaling [21]. Indeed, we observed an inhibitory effect of rapamycin on tunicamycin-induced upregulation of p-IRE1α, ATF4, and c-myc. However, the phosphorylation level of JNK was markedly enhanced, and the protein level for CHOP was not affected, as compared with tunicamycin single treated cells, which was inconsistence with previous studies [18, 20, 21].

The reason for this discrepancy is not clear based on data provided herein. This paradoxical observation might be explained by the following possibilities. First, ER stress is an adaptive response to unfavorable conditions by inducing ER-associated protein degradation (ERAD) signaling pathway to eliminate misfolded proteins and enhancing the capacity for protein folding and modification, in which new protein synthesis is required [1, 3] . Rapamycin exposure led to reduced abundance for proteins involved in this process, thus contributing to a protein overload in ER due to a decreased capacity to cope with unfolded proteins, ultimately leading to enhanced apoptosis signaling to maintain intracellular homeostasis. Second, JNK is a classic apoptotic protein activated by IRE1α under ER stress condition. In our study, rapamycin treatment led to decreased protein levels for IRE1α, but the protein phosphorylation for JNK was enhanced, instead of being attenuated. This unexpected result indicated the existence of IRE1α-independent regulation on JNK activation. Actually, in a recent study, Win et al. demonstrated that JNK can be activated in IRE1α-independent manner by interacting with Sab, a mitochondrial protein, and resulted in impaired mitochondrial respiration, enhanced reactive oxygen species production, and cell death in response to ER stress [35]. Rapamycin might enhance the interaction between JNK and Sab, thus, leading to increased cell death and intestinal barrier dysfunction, which is independent of IRE11α. Moreover, we found that rapamycin treatment led to a marked decrease in Bcl-2, an anti-apoptotic protein, in the presence of both rapamycin and tunicamycin, indicating an involvement of mitochondrial dysfunction in this process. Analysis of activity of enzymes involved in mitochondrial respiration chain and production of reactive oxygen species will be helpful to address this question. Even though mechanisms responsible for the ER stress-independent regulation of CHOP and JNK is currently unknown, enhanced JNK protein level and decreased Bcl-2 acted together to increase apoptosis in the combination treatment. Experiments are needed to uncover the underlying regulatory mechanisms. This line of basic research will help to prevent or ameliorate intestinal dysfunction in animals that often occurs under practical feeding and production conditions on farms [36].

## Conclusions

In conclusion, results of this study indicated that tunicamycin exposure led to ER stress-associated cell death which was accompanied by activation of mTORC1 signaling. Inhibition of mTORC1 by rapamycin resulted in enhanced cell death and increased intestinal permeability, suggesting a feed-back loop regulation on ER stress by mTORC1 signaling. Further study showed that JNK is a critical protein associated with cell death effect. More studies are required to elucidate underlying mechanisms, which might advance our understanding of the complex interaction between mTORC1 and ER stress signaling in intestinal epithelial cells.

## Abbreviations

4EBP1: Eukaryotic translation initiation factor 4E binding protein 1
ATF4: Activating transcription factor 4
ATF6: Activating transcription factor 6
Bcl-2: B-cell leukaemia/lymphoma 2
BiP: Binding immunoglobulin protein
CHOP: C/EBP homologous protein
DDIT3: DNA damage-inducible transcript 3
ER: Endoplasmic reticulum
ERK1/2: Extracellular signal-regulated kinase 1/2
IRE1: Inositol-requiring enzyme 1
JNK: C-Jun N-terminal kinase
mTORC1: Mechanistic target of rapamycin complex 1
p70S6K: 70 kDa ribosomal protein S6 kinase
PERK: Protein kinase RNA-like ER kinase
TEER: Trans epithelial electrical resistance
UPR: Unfolded protein response.
XBP1s: Spliced X-box binding protein 1
